# Human migration is important in the international spread of exotic *Salmonella* serovars in animal and human populations

**DOI:** 10.1017/S0950268813003075

**Published:** 2013-12-16

**Authors:** J. B. IVESON, S. D. BRADSHAW, R. A. HOW, D. W. SMITH

**Affiliations:** 1 PathWest Laboratory Medicine WA, QE11 Medical Centre Site, Nedlands, WA, Australia; 2 School of Animal Biology and Centre for Native Animal Research, University of Western Australia, Perth, WA, Australia; 3 School of Anatomy, Physiology and Human Biology, University of Western Australia, Perth, WA, Australia

**Keywords:** ‘Humanoses’, infections, native and exotic *Salmonella*, wildlife, zoonoses

## Abstract

The exposure of indigenous humans and native fauna in Australia and the Wallacea zoogeographical region of Indonesia to exotic *Salmonella* serovars commenced during the colonial period and has accelerated with urbanization and international travel. In this study, the distribution and prevalence of exotic *Salmonella* serovars are mapped to assess the extent to which introduced infections are invading native wildlife in areas of high natural biodiversity under threat from expanding human activity. The major exotic *Salmonella* serovars, Bovismorbificans, Derby, Javiana, Newport, Panama, Saintpaul and Typhimurium, isolated from wildlife on populated coastal islands in southern temperate areas of Western Australia, were mostly absent from reptiles and native mammals in less populated tropical areas of the state. They were also not recorded on the uninhabited Mitchell Plateau or islands of the Bonaparte Archipelago, adjacent to south-eastern Indonesia. Exotic serovars were, however, isolated in wildlife on 14/17 islands sampled in the Wallacea region of Indonesia and several islands off the west coast of Perth. Increases in international tourism, involving islands such as Bali, have resulted in the isolation of a high proportion of exotic serovar infections suggesting that densely populated island resorts in the Asian region are acting as staging posts for the interchange of *Salmonella* infections between tropical and temperate regions.

## INTRODUCTION

Prior to expeditions from the Northern Hemisphere and settlement by Europeans, the hunter-gatherer and other indigenous inhabitants of the Indo-Australian Archipelago and Australian sub-continent were exposed to only naturally occurring reservoirs of zoonotic infections evolving in native host species. In a limited study of *Salmonella* infections in reptiles in tropical areas of Western Australia (WA) [1] it was suggested that regional differences in the frequency of exotic serovars in humans and native fauna in WA were caused by the variable impact of European settlement. Both in the sparsely populated tropical areas of the state, as well as the more developed southern temperate coastal region, exotic serovars had spilled over into wildlife. These serovars were pre-adapted to the food chains of humans and introduced with domesticated animals translocated from the Northern Hemisphere [2].

These changes in the natural biogeography and epidemiology of *Salmonella* infections in indigenous humans and native fauna commenced during the colonial period. However, the introduction of exotic serovars is not confined to tropical and temperate regions of southern continents, since *Salmonella* serovars, active in the global epidemiology of salmonellosis, have recently been isolated from penguin and seal colonies exposed to human activities in the Antarctic region [3, 4].

*Salmonella* serovars classified as exotic infections in humans and animals in Australia, typically belong to antigenic groups B–E of the Kauffmann–White scheme, and have high international frequency quotients (QF), devised by Kelterborn [5]. They are a group of serovars prevalent in the Northern Hemisphere and associated in WA with European-style urban settlement and agricultural ecosystems. They are usually absent from native fauna in areas of less-disturbed natural habitat. Native salmonellae are defined as serovars falling in uncommon antigenic groups in wildlife in natural habitats that have not been disturbed by European-style settlement or agriculture. In tropical areas of WA, native serovars are prevalent in reptiles and native mammals and their persistence in the indigenous communities is linked to the traditional food customs of Aboriginal peoples exposed to the infected wildlife.

Native vertebrates on the islands of Java, Bali, and the Wallacea region [6], were first exposed to Europeans and introduced *Salmonella* infections at coastal trading posts established and operated by the Dutch, Portuguese and British in the 17th century. Colonization by the Dutch continued until Indonesia achieved independence in 1948. In WA, European settlement and introduced food culture commenced in temperate areas of the state in 1827, and the tropical East Kimberley region in 1890, when free-range cattle were introduced to the Ord River area. The impact on wildlife of the damming of the Ord River, and subsequent irrigation farming, dates from 1970.

*Salmonella* infections acquired by humans from native animals in Australia and Wallacea are not confined to tropical areas, and have occurred in residents and visitors exposed to droppings and close contact with quokkas (*Setonix brachyurus*) on Rottnest Island in WA, during peak summer holiday periods [7]. The island has been permanently settled since 1831, and now presents as a grossly disturbed environment where large numbers of animals infected with both native and exotic *Salmonella* serovars interact with humans in settlements, camping areas and tourist stops [8]. The number of quokkas in close contact with humans has been estimated to exceed 600 individuals (7·5% of the total population) with an average rate of infection in tagged animals monitored in settlement areas and tourist stops of 40%.

Tropical areas of Australia and Wallacea are of world heritage importance and are under increasing threat from expanding human populations and agriculture, mining of natural resources and international tourism. In 1986 over 400 000 Australians holidayed in Asian countries, the majority in Bali at international tourist resorts. By 2000, the number of Australian visitors to Bali had doubled and in that year the National Enteric Pathogens Surveillance Scheme (NEPSS) reported 514 (8%) *Salmonella* cases as infections in Australians acquired overseas; 167 (32%) of these were acquired in Bali. Conservation of natural habitat and maintenance of microbiological integrity in terrestrial and marine fauna is thus an urgent priority in the management of zoonotic infections in wildlife populations and the prevention of public health problems [9, 10].

There is extensive literature on salmonellosis as a zoonotic infection of medical and economic importance for humans and their livestock with wildlife, particularly reptiles, being considered as one of the primary sources of infection [11–14]. Less attention, however, has been focused on the reverse problem: that of the spillover of *Salmonella* serovars from humans and domestic animals into wildlife [10, 15]. Our hypothesis in this study is that exotic serovars, of Northern-Hemisphere origin, are gradually invading wildlife in previously pristine environments as human activity in these areas increases. As a test of this hypothesis, we compared the ratio of exotic to native serovars in wildlife in a variety of locations, varying in level of development and duration of human activity, in tropical and temperate regions of WA, and in 17 islands within the Wallacea region of Indonesia close to Australia.

The aims of the present study were thus fourfold: (*a*) to document the occurrence and distribution of *Salmonella* serovars, both native or exotic, as infections in humans and native wildlife in tropical and temperate regions of WA and the islands of Wallacea, (*b*) to evaluate the extent to which human activity has facilitated the infection of native wildlife in WA and the islands of Wallacea by exotic *Salmonella* serovars originating in the Northern Hemisphere, (*c*) to assess the usefulness of serovars as microbiological indicators of ecosystem disruption in areas of high natural biodiversity under threat from expanding human activity and, (*d*) to document exotic *Salmonella* infections currently entering WA from tourists after holidaying in Bali.

## MATERIALS AND METHODS

### Collecting locations and samples

**Fig. 1.**
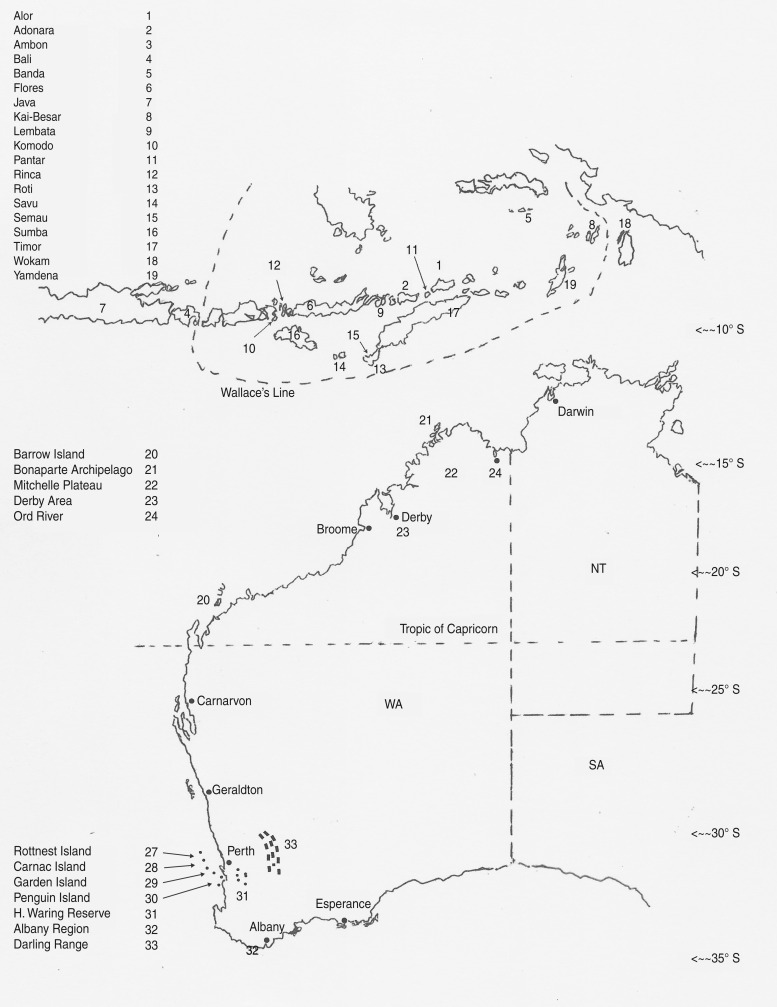
Sampling locations in the Wallacea region of Indonesia and north-western Australia and on offshore islands and the mainland in south-western Western Australia.

Case totals for *Salmonella* infections in humans over the period 1985–2000 inclusive, were provided by the NEPSS, from the national database [16], and comprised primary infections with single and multiple serovars in the Perth metropolitan region, Kimberley region of WA, Darwin region of the Northern Territory (NT), and notifications of infections in WA residents, acquired during holidays in Bali. Samples from wildlife (small mammals, snakes, lizards, frogs) were collected from 17 islands in the Wallacea region of Indonesia from 1989 to 1993 (Adonara, November 1989; Alor, April 1991; Ambon, May 1993; Banda, September 1992; Flores, October 1989, May 1990; Kai Besar, September 1992; Komodo, May 1990; Lembata, November 1989; Pantar, April 1991; Rinca, May 1990; Roti, October 1990; Savu, October 1990; Semau, May 1990, April 1991; Sumba, June 1989; Wokam, September 1992; Yamdena, April 1993; Timor May 1989, October 1990) plus from the island of Bali (October 1991), falling within the period when human infections were recorded. Eleven islands were sampled off the WA coast over the same time period, including the tropical Kimberley region and Bonaparte Archipelago (Fig. 1). Samples were collected strategically, targeting vertebrate species solely, especially those with a carnivorous diet and thus high up the food chain. Few birds were trapped in the Indonesian islands and thus have not been included in the analysis.

The WA mainland has an area of 2 526 786 km^2^ and extends from latitude 13^o^ S in the north to 35^o^ S in the south with a corresponding range in climate from tropical and subtropical in the north to temperate in the south. Inshore islands are numerous and account for a further 3089 km^2^, many of which harbour species of wildlife that are now extinct or endangered on the mainland. The aim of the study was to compare exotic *Salmonella* infections in wildlife (vertebrates, including small mammals, birds, reptiles and frogs) living in heavily urbanized areas in the southwest of WA with those in remote north-western regions of the state (Pilbara, Bonaparte Archipelago, Kimberleys) and the string of Indonesian islands close to the northern coast of Australia that form Wallacea. WA has a population of 2·43 million, 78% of whom live in the capital city, Perth (31^o^ 56′ S, 115^o^ 50′ E). The rest of the state is relatively sparsely populated. Animals sampled and their locations in WA are listed in Supplementary Table S1 (available online).

### Samples from wildlife on metropolitan islands close to Perth in WA

Commencing in 1970, a total of 4668 rectal swabs and occasional faecal and pouch swabs were collected in SCB transport broth from quokkas on Rottnest Island (32° 0·5′ S, 115° 30·1′ E) in settlement precincts, camping areas, a tourist stop, rubbish-tip site and the less-disturbed western end of the island. Forty cloacal swabs from King's skinks (*Egernia kingii*) and bobtail lizards (*Tiliqua rugosa*) and 20 swabs from individual snakes (dugites, *Pseudonaja affinis*) were collected mainly on roadsides and pathways away from settlements. Rectal swab samples and occasionally pouch swabs were also collected from 514 quokkas captured on Rottnest Island during an attempt to resettle marsupials free of exotic infections in the 283 ha Harry Waring Nature Marsupial Reserve, on the adjacent mainland [17].

On nearby Carnac Island Reserve (32° 7′ 22″ S, 115° 39′ 49″ E), cloacal swabs were collected over the period 1969–2000 by Health and Wildlife Authorities from 160 King's skinks, 48 tiger snakes (*Notechis scutatus*), 417 seagulls (*Chroicocephalus novaehollandiae*) and fresh faeces from Australian sea lions (*Neophoca cinerea*). Samples were also collected from 102 King's skinks on Penguin Island (32^o^ 18′ 21″ S, 115^o^ 41′ 27″ E). On adjacent Garden Island (32^o^ 13′ 13″ S, 115^o^ 41′ 17″ E), cloacal swabs were collected over the period 1988–2000 from 426 tammar wallabies (*Macropus eugenii*), six tiger snakes, a carpet python (*Morelia spilota imbricata*) and six skinks (*Tiliqua rugosa*).

### Native animals in the north-western Pilbara and Kimberley regions of WA

On the less-disturbed Barrow Island Class A Nature Reserve in the Pilbara (20^o^ 47′ 57″ S, 115^o^ 24′ 18″ E) over the period 1994–2000 rectal swabs were collected from 241 Spectacled hare-wallabies (*Lagorchestes conspicillatus*), occasional possums (*Trichosurus arnhemensis*), euro kangaroos (*Macropus robustus isabellinus*), burrowing bettongs (*Bettongia lesueur*) and cloacal swabs from 152 lizards and eight snakes. Cloacal swabs were also collected in the Kimberley region, from 33 reptiles and seven native mammals on the uninhabited Mitchell Plateau, islands in the Bonaparte Archipelago (Kimberley Islands), and from 46 reptiles in farming areas irrigated from the Ord River Dam over the period 1982–2000. A total of 1281 rectal swabs was collected, mainly from native mammals, and mostly in areas of natural habitat on the Mitchell Plateau [18]. The capture, handling and sampling of native vertebrates adhered to National Health & Medical Research Council guidelines and the Animal Ethics Committee of the University of Western Australia.

### Samples from native vertebrates in south-eastern Wallacea

During the period May 1989 to May 1993, 826 cloacal swab samples were collected from 57 species of reptiles and 634 rectal swabs from 65 species of native mammals [19]. Samples were also collected on the island of Bali and islands in southern and eastern Wallacea, including Aru and Timor Islands, the former close to New Guinea and the latter nearest to northern Australia (Fig. 1).

### Transport and culture procedures

Procedures followed those detailed in [3] and rectal swab samples from native mammals, and cloacal swabs from reptiles, were transported at ambient temperatures in strontium chloride B (SCB) broth and inoculated into SCB broth incubated at 43°C. Faecal samples from humans collected in buffered glycerol saline [20], and swabs from native fauna were cultured direct on deoxycholate-citrate (DC), bismuth-sulphate (BS) agar. Biochemical screening of up to 10 suspect colonies from plates was performed using Iveson's single tube GLISSUDA medium. Serum inhibition procedures were also used to increase isolations of different serovars [21]. Isolations of serovars and isolates classed in uncommon antigenic groups, were confirmed or identified by the Australian *Salmonella* Reference Centre (ASRC), Adelaide, SA, and new serovars listed in annual supplements of the Kauffmann–White scheme and catalogue of first isolations compiled by Kelterborn [22].

### Classification of serovars

Serovars classified as ‘exotic’ are cosmopolitan serovars, isolated initially from cattle, sheep, pigs, poultry, humans and effluents in the Northern Hemisphere, with the majority classed in subgenera I and antigenic groups B–E [5, 22]. Serovars classified here as native occur mostly in uncommon somatic groups F–Z continuing with numbers to 67 in the 2009 Kauffmann–White scheme [23]. Many of these serovars are host specific, particularly in reptiles [22].

### Statistical analyses

The analyses of serotype assemblage data for various categories were performed using the PRIMER v. 6 computer program [24] with the PERMANOVA+ add-on [25]. Numbers of serotypes were first standardized to the total, which transforms each assemblage count for each sample into a relative percentage, thus alleviating disproportionate sampling efforts. These percentages were then square-root-transformed to limit the impact of highly dominant types before being analysed using a Bray–Curtis similarity matrix with a Simprof test to determine significantly different assemblage groups. The similarity matrix allows each sample to be compared to every other sample based on the number of shared serovars; the matrix is then subjected to non-metric multidimensional scaling to indicate relationships and identify significant assemblages in two-dimensional space, with stress levels of the associated plots presented. Statistical analysis of differences in the ratio of exotic to native (E:N) serovars from the various sites was conducted using *χ*^2^ tests for heterogeneity with Statistix 9.0 (Softonic, Spain).

## RESULTS

Exotic and native serovars isolated from humans are shown in Table 1, with isolations listed for Perth and Darwin, the capital cities of WA and the NT, respectively; from the north-western Kimberley region of WA and isolations from tourists returning to WA after holidaying on the Indonesian island of Bali. Statistically, the ratio of E:N serovars does not differ from unity, except in the case of people returning from Bali where the ratio of 3·0 was significantly higher than in the other sites (*χ*^2^ = 9·36, *P* = 0·024). Enteritidis was by far the most common serovar in the Bali group with 258 isolations, followed by Hadar and Agona. Actual infection rates cannot meaningfully be calculated from human data, as samples are invariably collected from persons presenting with symptoms and thus do not constitute a random sample of the population. Nonetheless, from a total of 616 persons returning to Perth after holidaying in Bali presenting with intestinal complaints, 585 (95%), were positive for exotic *Salmonella* serovars. The frequency of isolation of exotic serovars in hospital admissions was lower in Perth than in persons returning from Bali (79% *vs.* 95·8%, *χ*^2^ = 87·22, *P* < 0·0001) and much lower in Darwin, the capital of NT (42%, *P* < 0·0001) and the Kimberleys (35%, *P* < 0·0001).

**Table 1.** Exotic and native *Salmonella* serovars, and their ratio, with isolations from humans in Perth, the Kimberley region of Western Australia, Darwin and from tourists returning to Perth from the Indonesian island of Bali

Serovars	Perth	Kimberley	Darwin	Bali	Antigenic group
**Exotic**					
Paratyphi A	9	0	1	1	A
Agona	64	4	18	29	B
Bredeney	39	25	12	0	B
Derby	43	1	2	12	B
Heidelberg	23	4	9	7	B
Java	113	41	82	13	B
Paratyphi B	5	2	1	2	B
Reading	1	5	20	0	B
Saintpaul	363	249	196	2	B
Schwarzengrund	9	1	2	2	B
II sofia	17	4	6	0	B
Stanley	38	0	7	14	B
Typhimurium	3407	115	137	13	B
Choleraesuis*	133	2	1	0	C1
Infantis	150	81	63	8	C1
Livingstone	17	5	3	1	C1
Mbandaka	62	2	25	6	C1
Montevideo	20	6	9	3	C1
Ohio	10	3	6	0	C1
Singapore	126	0	17	0	C1
Tennessee	92	134	42	0	C1
Thompson	9	0	5	5	C1
Virchow	83	3	21	12	C1
Blockley	20	1	2	15	C2
Bovismorbificans	127	18	9	1	C2
Emek	7	0	0	17	C2
Haardt	8	0	2	2	C2
Hadar	24	2	6	97	C2
Newport	191	10	19	5	C2
Corvallis	8	0	0	0	C3
Berta	22	0	2	22	D1
Enteritidis	81	6	19	258	D1
Javiana	21	1	1	11	D1
Panama	3	0	10	1	D1
Typhi	27	2	0	2	D1
Give	32	56	20	0	E1
Weltevreden	24	22	106	10	E1
Krefeld	6	1	1	1	E4
Kentucky	0	0	0	14	G
Cerro	57	2	7	0	K
No. of exotic serovars isolated	**39**	**30**	**36**	**30**	
**Native**					
Abony	6	48	3	0	B
Ball	4	16	122	0	B
Chester	263	169	146	0	B
Birkenhead	20	0	1	0	C1
Muenchen	276	166	50	0	C1
Oranienburg	191	125	72	0	C1
Oslo	6	0	34	5	C1
Potsdam	15	13	11	0	C1
Kottbus	7	3	6	1	C2
Litchfield	32	56	102	6	C2
Eastbourne	13	148	37	1	D1
Anatum var. 15+	172	183	84	5	E1
Ohlstedt	32	3	16	0	E1
Orion	50	35	13	0	E1
Senftenberg	37	195	32	5	E4
Rubislaw	9	48	28	0	F
Havana	91	170	68	1	G
Poona	9	62	13	0	G
Bahrenfeld	8	14	25	0	H
Charity	2	8	0	0	H
Hvittingfoss	5	43	23	1	I
Orientalis	12	0	13	0	I
Welikade	6	101	37	0	I
Jangwani	3	19	11	0	J
Kinondoni	0	1	49	0	J
Blukwa	20	4	0	0	K
II wandsbek	11	64	14	0	L
Urbana	8	56	32	0	N
Zehlendorf	0	10	0	0	N
Adelaide	96	71	38	4	O
Emmastad	0	8	12	0	P
Kimberley	0	18	1	0	P
Lansing	5	34	12	0	P
Wandsworth	7	107	38	2	Q
Bukavu	3	6	4	0	R
Johannesburg	3	2	8	0	R
II Fremantle	0	6	0	0	T
IV Houten	3	0	0	0	U
Treforest	0	5	6	0	51
IIIb Diarizona†	11	8	9	0	—
No. of native serovars isolated	**34**	**36**	**35**	**10**	
Exotic:native ratio	**1·15**	**0·83**	**1·03**	**3·0**	

* Now var. Kunzendorf australis.

† IIIb serovars not listed.

Exotic and native serovars from wildlife (reptiles and native mammals) collected from a range of habitats, both island and mainland, in the southwest of WA are given in Table 2. The ratio of E:N serovars is <1·0, ranging from 0·31 for Perth mainland to 0·91 for Carnac Island, with no significant differences (*χ*^2^ = 5·99, *P* = 0·2). Typhimurium was the major exotic serovar isolated from wildlife on Rottnest, Carnac and Penguin islands, with Javiana equally common on Rottnest. Derby and Bovismorbificans were also prevalent on Carnac Island. The E:N ratio for Garden Island was high at 0·50, but reflected the fact that only six native serovars were isolated, compared to 23 on both Rottnest and Carnac islands. Of some significance, is that 426 tammar wallabies were sampled on Garden Island over a period of a decade, without a single isolation of an exotic serovar, and only two isolations of native serovars.

**Table 2.** Exotic and Native *Salmonella* serovars, and their ratio, with isolations from wildlife, including lizards, snakes and native mammals collected from the Perth mainland and from Rottnest, Carnac, Penguin and Garden islands in the immediate vicinity of Perth, Western Australia

Serovars	Perth mainland	Rottnest Island	Carnac Island	Penguin Island	Garden Island	Antigenic Group
**Exotic**						
Agona	0	0	1	0	0	B
Bredeney	1	1	2	0	1	B
Derby	4	4	31	1	0	B
I 4,12:d:-	0	0	2	0	0	B
Java	0	0	1	0	0	B
Saintpaul	5	1	9	2	1	B
Typhimurium	5	81	67	9	0	B
Infantis	0	2	4	2	1	C1
Livingstone	0	0	4	0	0	C1
Montevideo	0	0	1	0	0	C1
Ohio	0	0	2	0	0	C1
Singapore	6	3	4	0	0	C1
Tennessee	1	0	5	1	0	C1
Bovismorbificans	4	7	11	4	0	C2
Newport	5	4	7	0	0	C2
Goldcoast	0	0	8	0	0	C2
Enteritidis	0	0	2	0	0	D1
Javiana	0	78	1	0	0	D1
Give	7	4	7	5	0	E1
Krefeld	0	0	1	0	0	E4
Kentucky	0	0	1	0	0	G
Cerro	2	0	0	0	0	K
No. of exotic serovars isolated	**10**	**10**	**22**	**7**	**3**	
**Native**						
Chester	16	89	7	1	1	B
Birkenhead	3	4	0	0	0	C1
Decatur	8	5	0	0	0	C1
Muenchen	20	439	34	3	0	C1
Oranienburg	13	94	4	0	0	C1
Potsdam	8	1	0	0	0	C1
Litchfield	0	0	2	0	0	C2
Eastbourne	0	0	0	3	0	D1
Anatum var. 15+	3	245	42	3	0	E1
Ohlstedt	6	0	0	0	0	E1
Orion	5	29	7	1	0	E1
Senftenberg	0	0	1	6	0	E4
Bullbay	2	0	0	0	0	F
Rubislaw	6	0	1	0	0	F
Havana	4	0	35	6	0	G
Poona	0	0	2	6	0	G
Rottnest	0	42	3	0	0	G
Bahrenfeld	6	39	1	1	0	H
Charity	4	0	5	4	0	H
II merseyside	0	0	6	18	0	I
Orientalis	41	86	3	0	0	I
II bleadon	8	7	0	0	0	J
Blukwa	15	4	0	0	0	K
Carnac	0	0	2	3	0	K
II Zeist	0	0	1	0	0	K
Jangwani	1	0	0	0	0	K
II Wandsbek	44	130	29	5	4	L
Adelaide	16	243	32	4	2	O
Champaign	6	0	0	0	0	Q
II Alsterdorf	20	8	1	0	0	R
II Sachenswald	1	0	0	0	0	R
Waycross	3	80	1	0	0	S
Coogee	1	0	0	0	0	T
II Fremantle	4	9	35	0	4	T
II Bunnik	9	6	2	0	0	U
IV Houten	12	3	0	0	0	U
Bootle	18	12	0	4	1	X
II 53:d:z42	2	3	0	0	0	53
IIIb Diarizona*	204	120	109	9	5	—
No. of native serovars isolated	**32**	**23**	**23**	**16**	**6**	
Exotic:native ratio	**0·31**	**0·43**	**0·95**	**0·5**	**0·5**	

* IIIb serovars not listed.

Table 3 lists exotic and native serovars isolated from wildlife (reptiles and native mammals) from the north-west of WA: from Barrow Island, some 80 km off the Pilbara coast of WA; from wildlife collected on the Kimberley mainland and from islands in the Bonaparte Archipelago, and from 17 Indonesian islands in the Wallacea region. A single exotic serovar, Give, was isolated from a perentie lizard (*Varanus giganteus*) on Barrow Island, with 22 isolations of native serovars, but no exotics from any of the endangered marsupial species on the island. A total of 10 exotic serovars was isolated from the Wallacea islands, with Newport being the most common with 33 isolations. The E:N ratio for the Wallacea islands was 0·38, significantly higher (*χ*^2^ = 11·57, *P* = 0·009) than for wildlife from the Kimberley Islands (0·0) and Barrow Island (0·05).

**Table 3.** Isolations of exotic and native *Salmonella* serovars, and their ratio, in lizards, snakes and native mammals collected on Barrow Island, the Kimberley mainland and Kimberley Islands off the north-west coast of Western Australia, and from islands in the Wallacea region of Indonesia

Serovars	Barrow Island	Kimberley mainland	Kimberley Islands	Wallacea Islands	Antigenic group
**Exotic**					
Java	0	2	0	4	B
Saintpaul	0	1	0	2	B
Stanley	0	0	0	4	B
Typhimurium	0	0	0	2	B
Singapore	0	2	0	0	C1
Tennessee	0	1	0	0	C1
Thompson	0	1	0	8	C1
Newport	0	0	0	33	C2
Enteritidis	0	2	0	11	D1
Javiana	0	0	0	4	D1
Panama	0	0	0	3	D1
Give	1	2	0	0	E1
Weltevreden	0	0	0	6	E1
No. of exotic serovars	**1**	**7**	**0**	**10**	
**Native**					
Abony	0	6	0	0	B
Ball	0	3	0	0	B
Chester	20	60	3	3	B
Lagos	0	0	0	8	B
Muenchen	5	45	1	15	C2
Oranienburg	11	40	3	17	C2
Oslo	0	1	0	8	C1
Potsdam	0	5	0	5	C1
Kalumburu	0	2	0	0	C2
Litchfield	0	5	0	2	C2
Mowanjum	0	2	0	1	C2
Bronx	0	8	0	0	C2
9,12:b:z57	0	9	0	0	D1
Bournemouth	0	2	0	0	D1
Eastbourne	0	17	0	20	D1
Amager	0	0	0	1	E1
Anatum var. 15+	0	5	0	0	E1
Lexington	2	2	0	1	E1
Ohlstedt	0	20	0	0	E1
Orion	5	6	0	0	E1
Senftenberg	0	2	0	0	E4
Bullbay	0	2	0	0	F
Chingola	0	0	0	6	F
Kisarawe	0	13	0	0	F
Rubislaw	16	74	1	26	F
Senegal	0	0	0	2	F
Havana	2	3	0	0	G
Poona	2	23	5	5	G
Rottnest	4	12	1	0	G
Bahrenfeld	38	61	2	0	H
Boecker	0	3	0	0	H
Charity	0	8	0	1	H
Horsham	0	4	0	0	H
Badagry	0	0	0	3	I
Hvittingfoss	10	39	1	13	I
Orientalis	0	0	0	13	I
Vancouver	0	0	0	15	I
Welikade	0	12	4	0	I
II bleadon	24	26	4	1	J
Jangwani	0	40	2	0	J
Kinondoni	0	3	0	0	J
II wandsbek	9	50	2	0	L
Brisbane	1	13	2	0	M
Angoda	0	21	0	0	N
Urbana	0	44	0	0	N
Ramatgan	0	5	0	0	N
Adelaide	6	21	4	0	O
Emmastad	0	4	0	0	P
Kimberley	0	1	0	0	P
Lansing	0	2	0	0	P
Champaign	0	1	1	2	Q
Wandsworth	0	5	0	2	Q
Bukavu	1	44	0	0	R
II alsterdorf	21	13	0	0	R
Waycross	0	0	0	6	S
II Fremantle	2	24	0	0	T
II Portbech	0	1	0	0	T
II Uphill	0	2	0	0	T
IV Houten	0	6	1	18	U
IV Lohbruegge	0	2	0	10	U
Bootle	17	45	1	0	X
Fitzroy	2	7	0	0	Y
Treforest	7	12	0	0	51
Ord	0	9	0	0	52
IIIb Diarizona*	18	116	10	175	—
No. of native serovars	**22**	**57**	**18**	**26**	
Exotic:native ratio	**0·05**	**0·12**	**0**	**0·38**	

* IIIb serovars not listed.

**Fig. 2:**
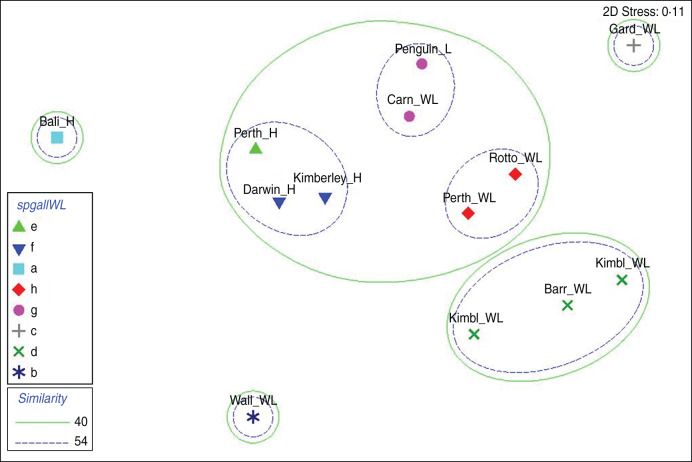
[*colour online*]. Relationship of total serovar assemblages in various faunal groups from different geographical locations using non-metric multidimensional scaling derived from a Bray–Curtis similarity matrix. Significantly different assemblages, determined using the Simprof test in the Primer software package, are indicated by different symbols. Spheres define important levels of similarity between clusters. Serovar assemblages in humans (_H) from different locations are indicated along with those in wildlife (_WL) from the same locations or Wallacean islands (Wall), Kimberley Islands (KimbI), Barrow Island (Barr), Garden Island (Gard), Rottnest Island (Rotto) and Carnac Island (Carn), as well as the lizards (_L) on Penguin Island.

**Fig. 3:**
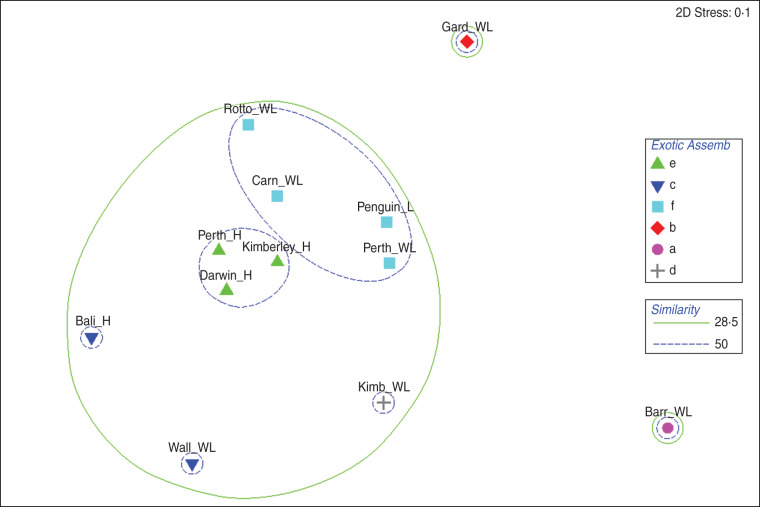
[*colour online*]. Relationship of exotic serovar assemblages in various faunal groups from different geographical locations using non-metric multidimensional scaling derived from a Bray–Curtis similarity matrix. Significantly different assemblages, determined using the Simprof test in the Primer software package, are indicated by different symbols. Spheres define important levels of similarity between clusters. Locations and groups as described in Figure 2.

**Fig. 4:**
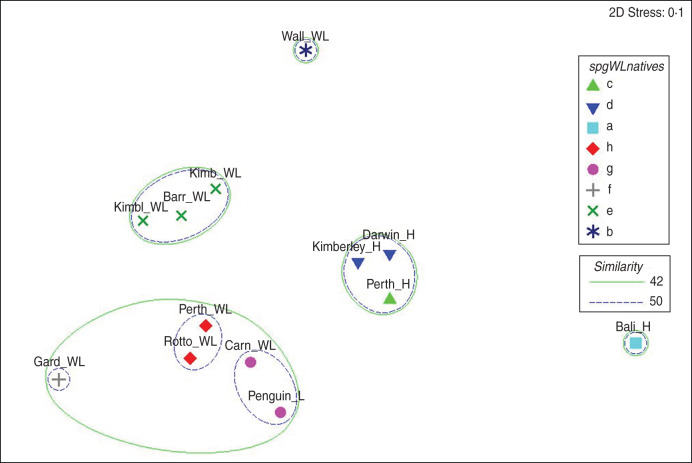
[*colour online*]. Relationship of native serovar assemblages in various faunal groups from different geographical locations using non-metric multidimensional scaling derived from a Bray–Curtis similarity matrix. Significantly different assemblages, determined using the Simprof test in the Primer software package, are indicated by different symbols. Spheres define important levels of similarity between clusters. Locations and groups as described in Figure 2.

Statistical relationships between exotic and native *Salmonella* serovars isolated from both humans and wildlife, when subjected to an analysis of assemblages, are shown in Figures 2–4. Serovar assemblages in human samples from Perth are significantly different from Kimberley and Darwin but still show a greater than 54% similarity. Assemblages in human samples from Bali are markedly different, having <40% similarity with the other human populations (Fig. 2). Carnac and Penguin island wildlife are statistically indistinguishable (over 54% similar), which is also the case with wildlife from Rottnest Island and the Perth mainland. All of the above (including human populations) are grouped together with >40% similarity, emphasizing the close link between the total suite of serovars shared by humans and wildlife in the Perth metropolitan region. These are quite distinct from wildlife on Barrow Island and in the north-west Kimberley region of the state which have similar serovar assemblages. The serovar assemblages in wildlife from Garden Island and Wallacea are significantly different from all other Australian locations and show no relationship with those in humans returning from Bali.

Relationships between exotic serovar assemblages only are shown in Figure 3.

The close grouping of serovar assemblages in humans from Perth, Darwin and the Kimberleys is evident with >50% similarity and the same is seen with wildlife from the Perth mainland and wildlife on Rottnest, Carnac and Penguin islands. What is also very clear is the grouping with 28·5% similarity of all these above locations with serovars from Bali humans, Wallacea wildlife and wildlife on the Kimberley mainland.

Garden and Barrow islands have very few exotic serovars which is reflected by small but very different assemblages. No exotic serovars were found in wildlife on the pristine Kimberley Islands, which are therefore not represented in the Figure 3.

**Fig. 5:**
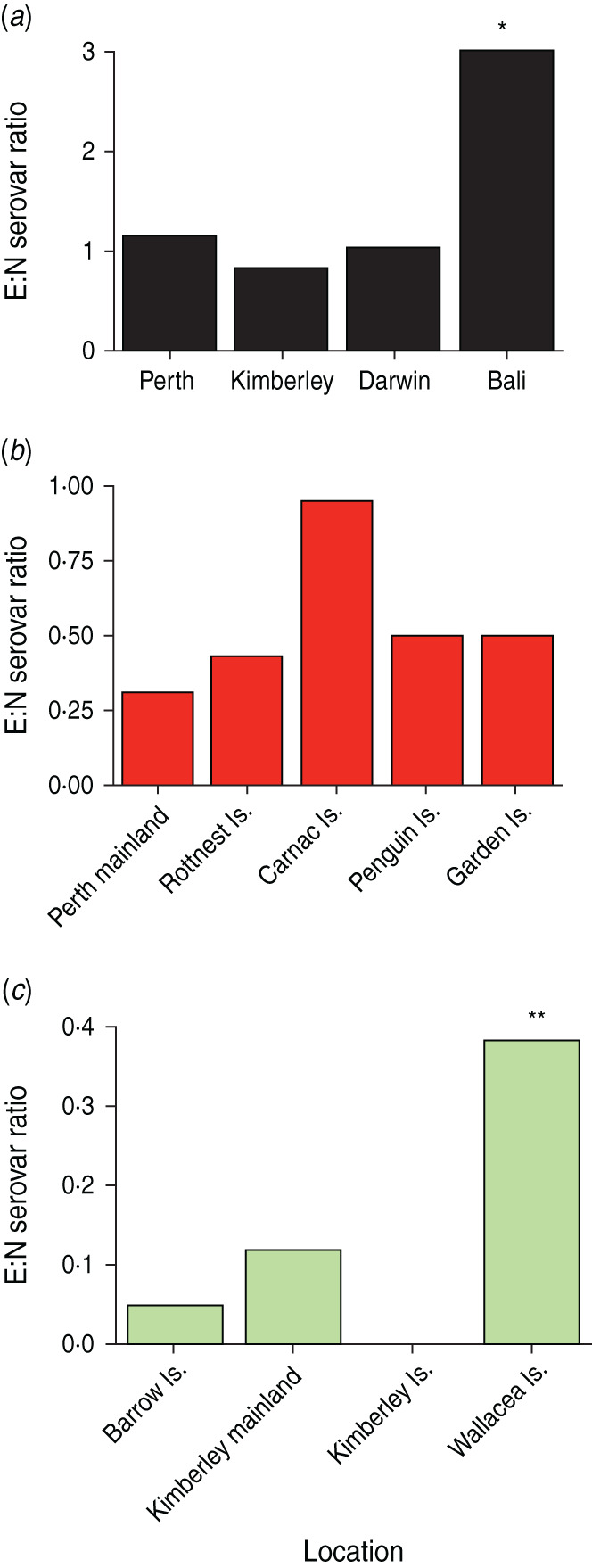
[*colour online*]. Variation in the ratio of exotic to native (E:N) serovars from listed locations with data from (*a*) humans, (*b*) wildlife in the south-west of Western Australia, (*c*) wildlife in the north-west of Western Australia and islands from the Wallacea region of Indonesia. Statistical significance: * *P* < 0·05 and ** *P* < 0·01.

The relationship of locations examining native serovars only is shown in Figure 4. Assemblages show similar groupings by location with the north-west wildlife samples (Barrow Island, Kimberley and Kimberley Islands) all significantly related (>50%) but distinct from assemblages found in south-western mainland and island populations, including Garden Island (<44%). Wildlife on Rottnest, and Perth mainland have similar serovar assemblages but significantly different from Carnac and Penguin islands. These assemblages differ from those on Garden Island and remain distinct from all human and other wildlife locations. Native serovars from humans returning from Bali were significantly different from those in Wallacea wildlife and from assemblages in humans living in Perth, Darwin and the Kimberleys. Variations in the ratio of E:N serovars for humans and wildlife in the various locations are shown in Figure 5. General details of wildlife sampled and numbers of serovars isolated in Indonesian wildlife are given in [19] with specific details of wildlife species sampled by island given in Supplementary Table S2.

## DISCUSSION

Studies over an extended period in WA have served to document the extent to which exotic *Salmonella* serovars gradually invade native wildlife with increasing development and urbanization of previously undisturbed island habitats and serve as ‘environmental barometers’ indicating degrading or degraded ecosystems [26–29]. Recent years have seen a growing interest in the problem of zoonoses and their impact on human health [9], as well as the converse of human-spread diseases impacting on wildlife (‘humanoses’) [10, 30–33]. The extent of the prevalence of *Salmonella*-infected wildlife worldwide is highlighted by recent studies documenting that 83% of *Salmonella enterica* serovars isolated from Antarctic penguins and seals were classifiable in high-frequency quotients for serovars prevalent in humans and domesticated animals in the Northern Hemisphere [3, 5].

Although many animals, especially reptiles, are thought to be ‘symptomless’ carriers of *Salmonella* serovars, there is evidence that salmonellosis can have a serious negative impact on some wildlife species. Antibodies against serovar Abortusovis, for example, were associated with reduced reproductive success in female alpine chamois [34] and mortality from botulism and salmonellosis in hot, dry summers is also commonly reported in water birds [35] and other wildlife [36, 37]. The present study builds on a preliminary study of *Salmonella* infections in reptiles in sparsely populated tropical areas of WA, where an unusual diversity of uncommon serovars, classed in numerically high antigenic groups, subspecies II and IIIb, and the first isolations of new serovars was recorded in the tropical Kimberley region [1]. It was also observed that serovar Typhimurium, prevalent in humans in the Northern Hemisphere [5] and southern areas of WA, was rarely isolated from indigenous human patients and was not recorded in reptiles in the Kimberley region [38]. A comprehensive study on the natural history of *Salmonella* infections in native mammals and reptiles on the Mitchell Plateau [18] confirmed the absence of serovar Typhimurium in native fauna in habitats not disturbed by Europeans or exotic animals.

A preliminary conference report of *Salmonella* and *Edwardsiella* infections in wildlife from 21 Indonesian islands between Java and Bali in the west, and Aru in the east, involved swabbing 1715 wildlife specimens from 187 species representing 59 families [19]. Over 70 serovars of *Salmonella enterica* were recorded with 54% coming from subspecies I, 0% subspecies II, 40% subspecies IIIb (*diarizonae*) and 6% subspecies IV. This contrasted with figures of 96%, 3%, 2% and 0·1% for isolates of the same subspecies in tropical Australia [38]. The present study, showing the close link between disturbed human-dominated locations and exotic Northern-Hemisphere serovars, confirms wildlife in undisturbed habitats being reservoirs of only native serovars.

An obvious limitation of our study arises from the fact that not all samples were taken at the same time and the database has been created by summing samples from relatively large time spans. In part, this is because we had no control over samples from humans, which were abstracted from the NEPSS database. Sampling of indigenous persons in the Wallacea islands was also not possible. Nonetheless, we believe that the data assembled provide a convincing picture of the significant invasion of Wallacean wildlife by exotic serovars of Northern Hemisphere origin. Of particular significance is the absence of these serovars in the Kimberley Islands, which have never been permanently settled by Europeans, and lie adjacent to the Indonesian islands, separated only by the Timor Sea (Fig. 1, Table 3).

The process by which exotic serovars displace, or occur as co-infections with native serovars, is best exemplified and our basic hypothesis tested by examining the situation of three metropolitan islands a few kilometres off the coast of Perth in WA: Rottnest, Carnac and Garden islands. All are small and have been subjected to human activity since the founding of the colony of WA in 1829 [39]. Carnac Island is now primarily populated by tiger snakes, lizards, house mice, Australian sea lions and a large nesting population of silver gulls; but it was, until recently, overrun with rabbits and had been used in the past to graze sheep, as a whaling station and as a prison for Indigenous Australians in 1837 [40]. Exotic infections now almost equal native serovars in the wildlife (21:23) with Typhimurium, Derby and Bovismorbificans predominating in snakes, King's skinks and seagulls. The much larger tourist destination of Rottnest Island, which supports a population (*∼*10–12 000) of marsupial quokka wallabies, has a lower E:N ratio of 0·43, with 10 exotic serovars dominated by Typhimurium and Javiana. Overall infection rates of exotic serovars in quokkas on Rottnest Island average ∼4% [28, 41, 42] but regional differences throughout the island are quite significant. Infection rates for exotic serovars in areas of heavy tourist visitation ranged from 27% to 52%, compared to just 3·9% in quokkas on the westernmost, and least disturbed, part of the island [26]. Garden Island, which is <3 km from Carnac Island, carries a large population of tammar wallabies but rigorous swabbing of over 400 individuals has failed to detect a single exotic serovar in this species. Garden Island differs, however, from the other two islands in being the property of the Federal Government of Australia, rather than the State Government of WA, and houses a large naval base with very limited access to the public. This accounts for the semi-pristine state of the marsupial wildlife on Garden Island compared to the disturbed situations on adjacent Rottnest and Carnac islands.

The situation in the islands of Wallacea at the present time is variable with some, such as Roti, Banda, Komodo, Kai-Besar and Semau, having wildlife heavily infected with multiple exotic serovars, whereas wildlife on other islands, such as Adonara, Flores, and Rinca, show no evidence of invasion by exotic serovars. Tourist visitation to Wallacean islands has expanded enormously in recent years climbing, for example, from 1000 visits per year in 1983 to 24 083 in 1996 for the island of Komodo [43]. We predict that Wallacean wildlife will become increasingly infected with cosmopolitan serovars with increasing visitations and thus function as reservoirs for zoonotic transmission to humans. The documenting of large numbers of WA residents returning from holidaying in Bali and presenting with infections of exotic *Salmonella* serovars, common to wildlife in the region, highlights what may be an emerging public health issue.

## CONCLUSION

The present study highlights the extent to which exotic serovars of *Salmonella enterica*, common to Northern-Hemisphere humans, domestic animals and poultry, progressively invade native wildlife in distant regions as contact with European civilization develops. In extreme cases, such as Rottnest and Carnac islands in WA, and the island of Bali in Indonesia, wildlife may also play a secondary role, serving as reservoirs for back-transmission of *Salmonella* to humans and thus pose a potential health hazard. We suggest that monitoring for exotic serovars of *Salmonella* in wildlife can function as an ‘early warning system’ for impending epidemics of salmonellosis. Increasing visitation and subsequent Europeanization of previously isolated island communities is likely to be followed by infection of their wildlife with exotic serovars, which subsequently can pose a zoonotic health hazard.

## Supplementary material

For supplementary material accompanying this paper visit https://doi.org/10.1017/S0950268813003075. click here to view supplementary material
