# Supplementary material for: Human migration is important in the international spread of exotic Salmonella serovars in animal and human populations
Source: Epidemiol Infect. 2013 Dec 16;142(11):2281–96. doi: 10.1017/S0950268813003075 (PMC4255324; doi:10.1017/S0950268813003075)
Supplement: Supplementary file 1 [file hygsup.zip › S0950268813003075sup001.doc]

Supplementary Table S1: List of sampling locations and wildlife tested for Salmonella infections (see text and Table S2 for specific names) * Metropolitan islands off the coast of Western Australia (WA)

| Location | Mammals | Reptiles | Amphibians | Birds | Exotic:Native Serovar ratio |
| --- | --- | --- | --- | --- | --- |
| Perth Mainland, WA | Bandicoot, echidna, Brush-tailed possum, Western grey kangaroo | Bobtail lizard, varanid, Tiger snake, dugite, Long-necked tortoise | Western green and golden bell frog, Slender tree frog | Seagulls, Black duck, Black swan | 0.31 |
| Rottnest Island, WA* | quokka | Dugite, Bobtail lizard, King skink |  |  | 0.43 |
| Carnac Island, WA* | Sea lions | Tiger snake, King skink |  | seagulls | 0.50 |
| Penguin Island, WA* |  | King skinks |  | Fairy penguins | 0.91 |
| Garden Island, WA* | Tammar wallaby | Tiger snake, Bobtail lizard, carpet python |  |  | 0.50 |
| Barrow Island, Pilbara region, WA | Spectacled hare wallaby, Northern possum, Burrowing bettong | Ringtail dragon, Perentie |  |  | 0.05 |
| Kimberley mainland, WA | Northern quoll, Northern bandicoot, planigale, Lakeland Downs mouse, Common rock rat | Ringtail dragon, Mountain devil, Perentie | Desert tree frog, Main’s frog, Desert toad |  | 0.12 |
| Kimberley Islands, WA | Common rock rat, Scaly-tailed possum, Black flying fox, Delicate mouse, Kimberley rock rat | Spiny-tailed monitor, Sand goanna, Black-palmed monitor, Timor monitor, Superb two-lined dragon, King brown snake, Yellow-faced whip snake |  |  | 0.0 |
| Wallacea islands, Indonesia | House shrews, Water rats, Native rats, Striped possum, Java pipistrelle | Flying dragon, skinks, geckoes, monitors, pythons, northern death adder, Pacific ground boa, Dog-faced water snake, White-lipped pit viper, Crab-eating water snake | Asian common toad, Crested toad, Common tree frog |  | 0.38 |
