# Supplementary material for: Human migration is important in the international spread of exotic Salmonella serovars in animal and human populations
Source: Epidemiol Infect. 2013 Dec 16;142(11):2281–96. doi: 10.1017/S0950268813003075 (PMC4255324; doi:10.1017/S0950268813003075)
Supplement: Supplementary file 1 [file hygsup.zip › S0950268813003075sup002.doc]

Supplementary Table S2 : Host species and isolations of exotic Salmonella serovars in lizards (L), snakes (S), frogs (F) and native mammals (NM) from islands in the Wallacea region of Indonesia, from Bali to Wokam .*

| **Island and Exotic:Native Serovar Ratio** | **Host** | **Serovar** | **Isolations** |
| --- | --- | --- | --- |
| Alor | *Broghammerus reticulatus (S)* | Enteritidis | 1 |
|  | *Lycodon aulicus (S)* | Weltevreden  Java | 1  1 |
|  | *Trimeresurus albolabris (S)* | Panama | 1 |
| *E:N Ratio* | 0.67 | | |
| Ambon | *Suncus murinus (NM)* | Newport  Enteritidis | 1  1 |
| *E:N Ratio* | 0.34 | | |
| Banda | *Candoia carinata (S)* | Enteritidis | 5 |
|  | *Suncus murinus (NM)* | Enteritidis, Weltevreden | 3  1 |
| *E:N Ratio* | 1.0 | | |
| Kai-Besar | *Acanthophis laevis (S)* | Newport | 1 |
|  | *Hydromys chrysogaster (NM)* | Enteritidis | 1 |
|  | *Suncus murinus (NM)* | Newport, Thompson,  Stanley,  Enteritidis,  Java,  Weltevreden | 1  1  2  1  1  1 |
|  | *Cyrtodactylus semenanjungensis (L)* | Enteritidis | 1 |
| *E:N Ratio* | 0.75 | | |
| Komodo | *Hemidactylus platyurus (L)* | Weltevreden | 1 |
|  | *Gekko gecko (L)* | Weltevreden | 1 |
| *E:N Ratio* | 1.0 | | |
| Lembata | *Cerberus rynchops (S)* | Newport | 1 |
|  | *Pipistrellus javianicus. (NM)* | Newport | 1 |
|  | *Suncus murinus (NM)* | Newport,  Panama,  Weltevreden | 1  2  1 |
|  | *Varanus salvator (L)* | Panama | 1 |
| *E:N Ratio* | 0.38 | | |
| Pantar | *Komodomys sp. (NM)* | Java | 1 |
| *E:N Ratio* | 0.5 | | |
| Roti | *Gekko gecko (L)* | Newport, | 2 |
|  | *Eutropis multifasciata (L)* | Weltevreden | 1 |
|  | *Hemidactylus brookii (L)* | Newport  Weltevreden | 3  1 |
| *E:N Ratio* | 2.0 | | |
| Savu | *Cerberus rynchops (S)*  *Hemidactylus frenatus (L)* | Newport  Weltevreden | 2  1 |
|  |  |  |  |
| *E:N Ratio* | 0.25 | | |
| Semau | *Coelognathus subradiata (S)* | Javiana | 1 |
|  | *Gekko gecko (L)* | Javiana | 1 |
|  | *Hemidactylus brookii (L)* | Weltevreden | 1 |
|  | *Fejervarya sp.(F)* | Newport | 1 |
|  | *Trimeresurus albolabris (S)* | Panama | 1 |
| *E:N Ratio* | 1.0 | | |
| Sumba | *Spenomorphus florensis (L)* | Javiana | 1 |
|  | *Coelognathus subradiata (S)* | Stanley | 1 |
|  | *Boiga cynodon (S)* | Panama | 1 |
|  | *Trimeresurus albolabris (S)* | Weltevreden | 1 |
| *E:N Ratio* | 0.6 | | |
| Wokam | *Litoria infrafrenata. (F)* | Thompson | 2 |
|  | *Rattus steini (NM)* | Thompson | 1 |
|  | *Dactylopsila trivirgata (NM)* | Thompson | 1 |
|  | *Hypsilurus sp. (L)*  *Varanus indicus (L)* | Thompson  Thompson | 1  1 |
| *E:N Ratio* | 0.5 | | |
| Yamdena | *Acanthophis laevis (S)* | Newport  Thompson | 1  1 |
|  | *Amphibolurus temporalis (L)* | Java | 1 |
|  | *Polypedates leucomystax (F)* | Java | 1 |
| *E:N Ratio* | 0.43 | | |
| Bali | *Duttaphrynus melanosticus (F)* | Virchow | 1 |
|  | *Ingerophrynus biporcatus (F)* | Blockley | 1 |
|  | *Dendrelophis pictus (S)* | Weltevreden | 1 |
| *E:N Ratio* | 1.0 | | |
| Timor | *Draco timoriensis (L)* | Newport | 1 |
|  | *Liasis mackloti (S)* | Newport | 1 |
|  | *Cerberus rynchops (S)* | Newport | 15 |
|  | *Fordonia leucobalia (S)* | Saintpaul | 2 |
|  | *Fejervarya cancrivora. (F)* | Thompson | 1 |
|  | *Polypedates leucomystax (F)* | Saintpaul | 1 |
|  | *Varanus timorensis (L)* | Javiana | 1 |
|  | *Trimeresurus albolabris (S)* | Typhimurium | 2 |
|  |  | Stanley | 1 |
|  |  | Newport | 2 |
| *E:N Ratio* | 0.4 | | |

*There were no isolations of exotic serovars from wildlife sampled on the following islands in Wallacea: Adonara, Flores and Rinca.
